# Pediatric health care professionals’ vaccine knowledge, awareness and attitude: a survey within the Italian Society of Pediatric Allergy and Immunology

**DOI:** 10.1186/s13052-021-01090-9

**Published:** 2021-09-09

**Authors:** Elisabetta Del Duca, Loredana Chini, Simona Graziani, Mayla Sgrulletti, Viviana Moschese, with the Italian Pediatric Immunology and Allergology Society (SIAIP) Vaccine Committee, V. Moschese, L. Chini, M. Sgrulletti, R. M. Dellepiane, B. Martire, M. Sangerardi, D. Montin, G. Ottaviano, C. Rizzo, M. Duse, G. Marseglia

**Affiliations:** 1 Pediatric Immunopathology and Allergology Unit, Policlinico Tor Vergata, University of Rome Tor Vergata, Viale Oxford 81, 00133, Rome, Italy; 2 PhD program in Immunology, Molecular Medicine and Applied Biotechnology, University of Rome Tor Vergata, Rome, Italy; 3 Department Saint Camillus International University of Health and Medical Sciences, Rome, Italy; 4 Department of Pediatrics, Fondazione IRCCS Ca’ Granda, Ospedale Maggiore Policlinico, Milan, Italy; 5 UOC of Pediatrics and Neonatology, “Monsignor A.R. Dimiccoli” Hospital, Barletta, Italy; 6 Unit of Pediatrics, “Policlinico-Giovanni XXIII” Hospital, University of Bari, Bari, Italy; 7 Department of Public Health and Pediatrics, Regina Margherita Children Hospital, University of Turin, Turin, Italy; 8 Pediatric Haematology, Milano-Bicocca University, Monza, Italy; 9 Innovation and Clinical Pathways Unit, Bambino Gesù Children’s Hospital, IRCCS, Rome, Italy; 10 Division of Pediatric Immunology and Rheumatology, Department of Pediatrics, Sapienza University of Rome, Rome, Italy; 11 Pediatric Clinic, Fondazione IRCCS Policlinico San Matteo, University of Pavia, Pavia, Italy

**Keywords:** Vaccine hesitancy, Vaccine knowledge, Vaccine attitudes, Pediatric health care professionals, Channel sources

## Abstract

**Background:**

Physicians play a key role in driving vaccine acceptance and their recommendations are crucial to address vaccine hesitancy. The aim of the study was to assess knowledge, awareness and attitude of Italian Pediatric Health Care Professionals (pHCPs) on vaccinations.

**Methods:**

An anonymous on-line questionnaire was developed within the Vaccine Committee of Italian Society of Pediatric Allergy and Immunology (SIAIP) and spontaneously completed by 231 Pediatricians and Pediatric Nurses (PN).

**Results:**

An accurate vaccine education was reported by 70% of pediatricians and 13% of PN but 11% of pediatricians versus 26% of PN consult social media instead of scientific sources for their vaccine update. The investigation on the pHCPs attitudes to vaccination in a personal and family setting highlights poor adherence to vaccinations. Only 63% of pediatricians versus 16% of PN (*p* < 0.0001) annually received the Flu vaccine. In their family setting 93% of pediatricians versus 51% of PN recommended all vaccinations (*p* < 0.0001). Anti-flu, anti-rotavirus, anti-zoster and anti-pneumococcal vaccines were not regularly recommended by all pHCPs due to doubts of uselessness (55% of pediatricians versus 40% of PN) and preference for “natural immunity” (44% of pediatricians versus 40% of PN).

**Conclusions:**

Our results indicate that pHCPs’ attitude and confidence in regards to vaccines remain suboptimal. Current COVID-19 pandemic and the rapid development of vaccines could increase vaccine hesitancy. Due to the documented pHCPs’ influence in the parental decision, educational interventions are needed to improve their level of knowledge and counselling skills in order to address parental vaccine hesitancy and to maintain continuity of immunization services.

## Background

The World Health Organization (WHO) recommends vaccine coverage threshold of 95% to prevent and control the circulation of viruses and bacteria in the community and to reach the so-called “herd immunity”. In order to contribute to the implementation of immunization programs in all countries an European Immunization Plan (European Region Vaccine Action Plan) has been developed [1]. In Italy, since 2005, the Ministry of Health has published the National Immunization Prevention Plan (PNPV) to support a uniform vaccination strategy across all regions; nevertheless, in the last decade, vaccination coverage showed a decline in almost every region of the country, suggesting low vaccine awareness and increased parental vaccine hesitancy.

For example, polio vaccine coverage rate fell from 96.1% in 2011 to 93.3% in 2016 at 24 months of age [2] and between 2013 and 2015 the vaccine coverage for measles, mumps and rubella (MMR) fell from 90 to 85%, at 24 months of age [2]. Furthermore, between January and December 2017, a severe measles outbreak occurred in Italy, with 4885 measles cases, including 4 deaths [3].

To increase vaccination rates, a novel National Vaccine Prevention Plan (PNPV 2017–2019) [4] extended the list of recommended vaccines and introduced new target populations. Vaccines against *Neisseria meningitidis B*, rotavirus and varicella were recommended for children; tetravalent meningococcal vaccine (ACWY135) and a booster dose of anti-polio for adolescents, as well as human papillomavirus vaccine (HPV) for male adolescents; last but not least conjugated (PCV) or polysaccharide pneumococcal vaccines (PPV) and zoster vaccination for subjects over 65 years old and at-risk categories. Moreover, such vaccines were included into the Essential Care Levels of Assistance (LEA) and information campaigns were promoted. To achieve the goals of the PNPV, an urgent Decree-Law (No.73 June 2017), subsequently converted into a law (No.119 July 2017) approved 10 mandatory vaccines, such as pertussis, measles, mumps, rubella, varicella, *Haemophilus influenzae type b*, diphtheria, tetanus, hepatitis B and polio vaccines [5]. According to the law, these vaccines are mandatory for school admission. In 2018, a slight vaccine coverage increase was observed in some Italian regions, but results were far from the WHO recommended threshold: among children at 24 month of age, vaccination coverage for diphtheria, polio, tetanus barely reached 95% whereas for hepatitis B it was still below 95%. Polio vaccine uptake exceeded 95% in 14 regions, whereas in 7 regions was less than 93% [2]. As reported in a recent Italian survey, parental vaccine hesitancy due to safety and efficacy is still high [6].

Several studies have underlined that physicians’ recommendation is a key determinant in the parental decision-making process to vaccinate their children [7], also a favorable parent opinion is strongly influenced by a favorable physician opinion [8, 9]. The aim of our study was to assess vaccine knowledge and awareness among Italian Pediatric Health Care Professionals (pHCPs) such as Pediatricians and Pediatric Nurses (PN), as well as their personal and family vaccination adherence, to draw a real picture of their attitude.

## Methods

The Vaccine Committee of the Italian Society of Pediatric Allergy and Immunology (SIAIP) has implemented an anonymous, online and multiple choice questionnaire for pediatricians and PN published on the official website of the Society (www.siaip.it). The questionnaire included 31 items consisting of three major sections and was delivered by Google Supplementary Modules. The first section contained questions to define respondents’ training in vaccinology and their channel sources for vaccine-related updates. The second section aimed to assess their perception on parental vaccine concerns and hesitancy, their attitude to discussing the vaccine issue with families as well as their awareness regarding vaccine uptake in childhood. The last section analyzed their personal and family vaccine attitudes.

One hundred and thirty seven pediatricians, belonging to the SIAIP and to the Italian Health System, and 94 PN spontaneously answered to the online survey.

Statistical analysis was performed using Fisher’s exact test and Chi-Square test with a *p* value ≤0.05 considered significant.

## Results

### pHCPs’ training in vaccinology and channel sources for vaccine update

The answers to the first section showed that 96/137 (70%) pediatricians compared to 12/94 (13%) PN considered their training appropriate (*p* < 0.0001). 109/137 (80%) pediatricians and 66/94 (70%) PN used the Italian Health Minister and the National Institute of Health (ISS) websites as well as scientific literature for their update. Conversely, 15/137 (11%) pediatricians and 24/94 (26%) PN consulted channel sources such as social networks, blogs or media.

### pHCPs’ perception of parents’ vaccine concern and hesitancy and the dialogue with families

One hundred ninety eight out of 231 (86%) pHCPs declared to perceive an increase in parental concern on vaccine safety/efficacy, with no significant differences between pediatricians and PN (data not shown).

**Fig. 1:**
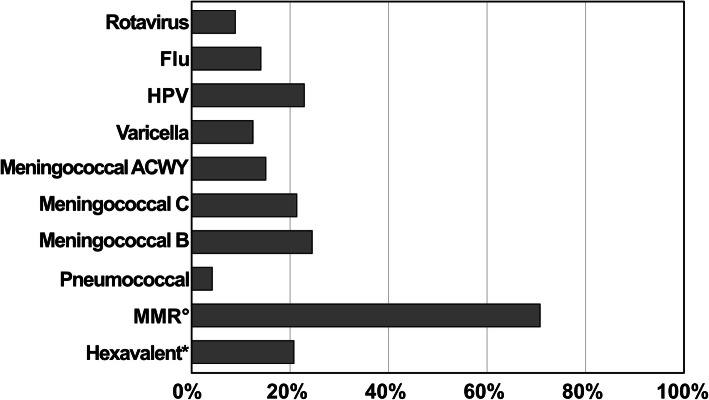
Parental concern and refusal to specific vaccines as reported by Pediatricians and Pediatric nurses (° Measles-Mumps-Rubella; * Polio-Difteria-Tetanus-Pertussis-Hepatitis B-*Haemophilus influenzae type b*)

Main concerns and related vaccines are shown in Table 1 and in Fig. 1, respectively.

**Table 1:** Main determinants of parental vaccine hesitancy referred by 198/231 Pediatric Health Care Professionals (pHCPs)

	Total 198	%
**Concern about vaccine adverse reactions**	145	73%
**Concern about development of autism spectrum disorders**	90	45%
**Concern about development of autoimmune diseases**	86	43%
**Doubts on usefulness and effectiveness of vaccines**	75	38%
**Concern about adjuvants adverse effects**	38	19%
**Concern about pain and stress induced by vaccination**	36	18%
**Preference for the development of natural immunity**	32	16%
**Concern about the risk to get the disease by the vaccination**	29	15%
**Lack of trust in pharmaceutical companies**	29	15%
**Too expensive**	23	12%

**Fig. 2:**
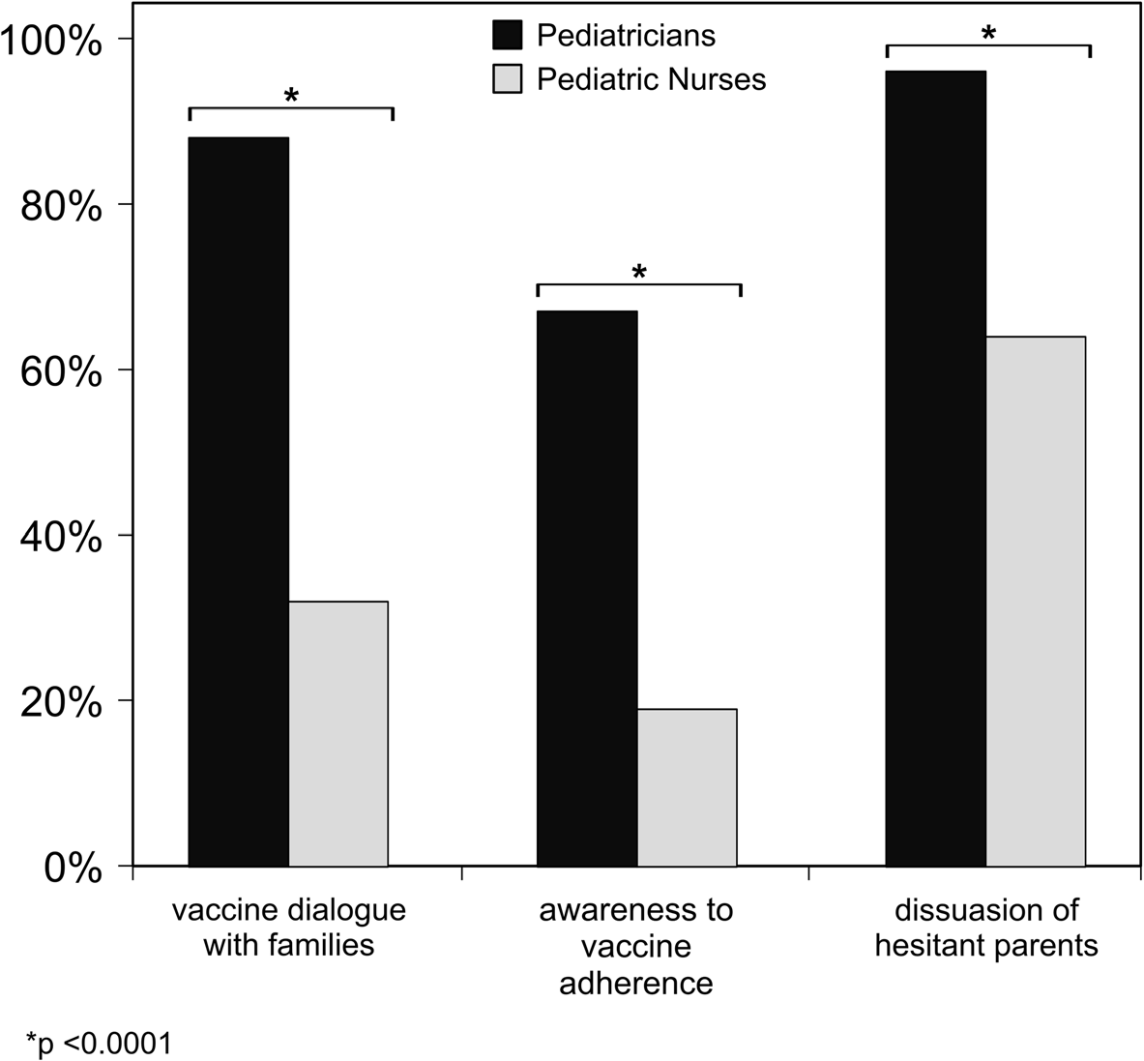
The behaviours of Pediatricians and Pediatric Nurses on parental vaccination making process with regard to vaccine dialogue with families, awareness to vaccine adherence and dissuasion of hesitant parents

A continuous vaccine communication with the families was reported by 121/137 (88%) pediatricians versus 30/94 (32%) PN (*p* < 0.0001) (Fig. 2).

Moreover, 92/137 (67%) pediatricians claimed to regularly verify children’s adherence to vaccine schedules versus 18/94 (19%) PN (*p* < 0.0001); also, 132/137 (96%) pediatricians versus 60/94 (64%) PN (*p* < 0.0001) tried to dissuade the hesitant parents (Fig. 2).

### Vaccine attitude of pediatric health care professionals in personal and family setting

**Fig. 3:**
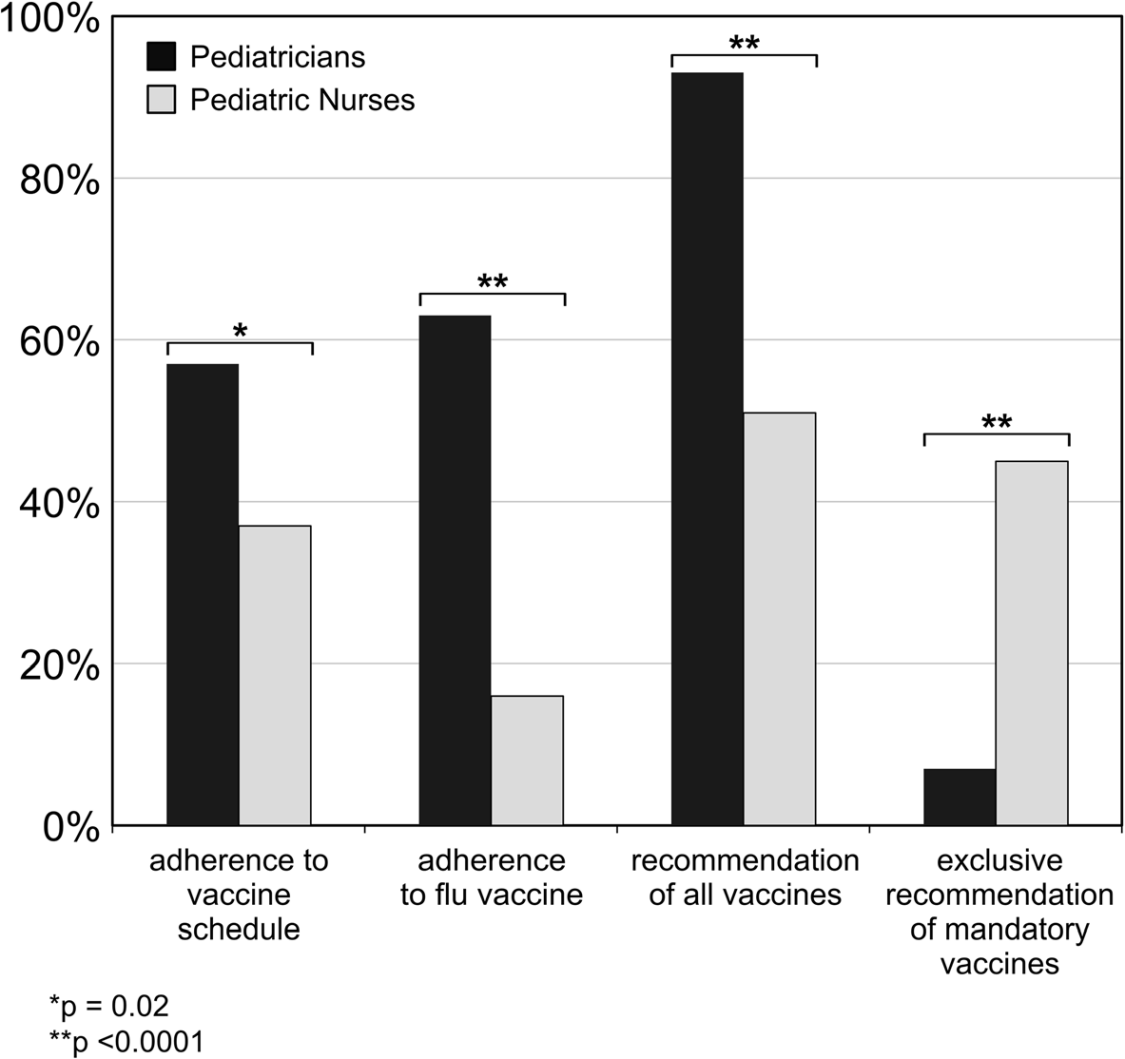
Personal and family vaccine attitude of Pediatricians and Pediatric Nurses for adherence to routine vaccine schedule, adherence to flu vaccine, recommendation of all vaccines and exclusive recommendation of mandatory vaccines in the family setting

Adherence to vaccination schedule has been reported to be constant in 78/137 (57%) pediatricians versus 35/94 (37%) PN (*p* = 0.02) (Fig. 3). On the question “*Do you yearly get flu vaccine?”* 86/137 (63%) pediatricians versus 15/94 (16%) PN replied “yes” (*p* < 0.0001) (Fig. 3).

In the family setting, 127/137 (93%) pediatricians and 48/94 (51%) PN promoted vaccine recommendations (*p* < 0.0001), with 10/137 (7%) pediatricians and 42/94 (45%) PN recommending only the mandatory vaccines (*p* < 0.0001) (Fig. 3).

Anti-flu, anti-rotavirus, anti-zoster and anti-pneumococcal vaccines were not regularly recommended. The main reasons for vaccine refusal were doubts of uselessness in 75/137 (55%) pediatricians versus 38/94 (40%) PN and preference for “natural” immunity in 60/137 (44%) pediatricians versus 38/94 (40%) PN. Moreover 4/94 (4%) PN declared No-vax position.

## Discussion

Vaccine hesitancy is an important issue that needs to be addressed to achieve effective control over vaccine-preventable diseases [10]. Parental concerns are mainly related to the lack of confidence on vaccine efficacy and safety, as well as to misinformation (fake news) regarding adverse effects. The latter includes, in particular, the presumed and disavowed link between vaccines and autism, allergies or autoimmune diseases [6, 11] and is derived from the growing opposition movements due to ideological reasons (no-vax) [11].

Among the possible reasons for vaccine hesitancy the lack of appropriate recommendations with simple and clear information by pHCPs could also play a role [12]. Therefore, univocal information and a strong family-HCP relationship based on awareness and dialogue are essential to address vaccine concerns and improve vaccine uptake [7, 8].

It is a fact that after the Decree-law of June and the law of July 2017, vaccine coverage against tetanus, polio, MMR, pneumococcal and meningococcal C, showed respectively a 0.9, 1.2, 4.4, 2.4 and 2.4% increase in the birth cohort of 2017 compared to 2016 [13]. Nevertheless, according to data reported by National Institute of Health, in Italy, between January 2018 and December 2018, measles cases were 2526, including 8 deaths [3], while those reported in 2019 were 1627 with more than half of them in Lombardy and Lazio regions. The average age of the affected patients was 30 years but the highest incidence was reported in children < 5 years of age. Indeed, among 174 cases including 64 children < 1 year (136.9 cases/1.000.000), 86% of patients were not vaccinated [3]. Of note, 96/1627 cases have been reported among health care providers. Between January and August 2020, measles cases were 101 in 12 regions with an average age of 33 years. Again, in 2020 the highest incidence has been reported in children < 5 years of age (4.6 cases/1000000) and five cases reported in children < 1 year of age (11.4 cases/1000000). 93% of all patients were not vaccinated with 12 cases among health care professionals. Although these data show a higher trend in vaccine coverage, in addition to legislative measures, other strategies are necessary to improve vaccine uptake [14]. From a cross-sectional survey aimed to estimate vaccine hesitancy and its determinants on 3130 parents of children aged 16–36 months, emerged the fact that, in Italy, safety concerns are the main reported reasons for refusing or interrupting vaccinations [6]. Accordingly, the majority of pHCPs respondents reported that doubts on safety and efficacy of vaccinations were the main reasons for parental refusal and hesitation. Inadequate and mostly negative information by media and websites have certainly increased parental vaccine-hesitancy. An analysis conducted on 153 YouTube videos on vaccination in children indicated that, in 50% of the cases, the information on immunization were not pro-vaccine and rather discordant with scientific literature; another observational study reported that YouTube videos that disapproved vaccination were the most shared by the viewers [15, 16]. The European Center for Disease Prevention and Control (ECDC) and other public health organizations [17, 18] published guidelines for health care professionals to provide them with tools to optimize the effectiveness of vaccine discussion with parents. In this regard, our data show that PN, compared to pediatricians, are less conscious to track children’s vaccine schedule and provide parents with factual information about vaccine programs. However, vaccine education and training was mostly considered inappropriate by pediatricians and PN during their academic and professional years. The observation that approximately 10% of pediatricians and 20% of PN consult social media rather than scientific sources for vaccine update, urges the need for improving educational and counselling skills among pHCPs. Studies from other WHO regions recommend vaccine education programs to increase health professional’s knowledge and awareness to enhance vaccination coverage [19–22].

In our study we found poor adherence of pediatricians and PN to vaccine schedule, since only 57% of pediatricians and 37% of PN respond to vaccine recommendations. In particular, only 63% of pediatricians and 16% of PN receive the annual flu vaccine. Things do not go better for PN in the family setting, since a significant proportion compared to pediatricians (45% versus 7%; *p* < 0,0001), recommend only selected vaccines due to their concerns on vaccine efficacy and their preference for the development of “natural” immunity. It goes without saying, this behaviour might affect parental decision- making process to vaccinate their children.

Although there are significant differences between pediatricians and PN, our current findings show that vaccine attitude and knowledge of pediatric health care professionals remains suboptimal mainly due to misinformation and concerns regarding vaccine safety and efficacy.

In Table 2 we indicate main implementation strategies to improve vaccination management among pHCPs. Educational themes represent a priority, but also communication skills and participatory practices should be encouraged. In fact counselling skills are key elements to tackle vaccine hesitancy as recently recommended by the National Institute of Health [23]. Last but not least, an appropriate environment with structural, administrative and digital supports would motivate health professionals and positively affect their efforts to sustain vaccine uptake.

**Table 2:** Main implementation strategies to improve Pediatric Health Care Professionals (pHCPs) knowledge and awareness

Provide appropriate vaccine knowledge and training on vaccinology during academic years	
Favor easy and free access for vaccine update	
Plan continuing educational interventions	
Ensure tools to empower communication skills and basic techniques of relationship	
Organize intersociety meetings to update of vaccine guidelines	
Qualify Vaccine Centers with appropriate structural and administrative supports	
Improve use of digital technology for vaccine programs	

We acknowledge that the assessment of pHCPs’ knowledge, awareness and attitude on a limited sample size should be complemented by further data collection to allow a more comprehensive generalizability of our findings. However, since the questionnaire was filled in by voluntary and anonymous pediatricians and PN that have a direct counseling role we believe that this survey may represent a useful evidence base of pHCPs’ vaccine confidence. Indeed, healthcare workers provide role modeling for preventive behaviors and the bulk of immunization strategies. Covid-19 pandemic makes such educational interventions even more urgent due to the occurrence of disruption or delay in routine vaccination programs with the risk of re-occurrence of vaccine preventable disease. Further, with the introduction of Covid-19 vaccine a clear and detailed information to healthcare workers is to be prioritized since a high rate of vaccine skepticism has been observed, including health care workers [24]. In this regard the Ministry of Health has issued a document with 2020–2021 vaccine recommendations. Flu vaccination has been offered actively and free of charge to all children from 6 months to 6 years of age and recommended in all pediatricians and pregnant women.

To strengthen vaccine uptake distinct international and national organizations as well as scientific societies including the Italian Society of Pediatric Allergy and Immunology (SIAIP), can give a significant contribution to patrol clear and simple information to all stakeholders. In this regard in October 2019 the European Academy of Allergy and Clinical Immunology (EAACI) has produced a video to spread vaccination culture worldwide [25]. Furthermore, in March 2020, the WHO has published a document with guiding principles to maintain continuity of immunization services [26] and, in June 2020, the SIAIP provided a Consensus Statement to offer a rationale to help guide decision-making in the management of children and adolescents with allergic or immunologic diseases [27].

## Conclusions

Although the sample size was limited, our data show the urgent need to design and plan educational interventions to improve Health Care Professionals’ vaccine knowledge and to ensure optimal acquisition of communication skills for vaccine uptake. This is particularly true at Covid-19 pandemic time where key protection of children and the entire community against serious vaccine-preventable disease can be governed by inclusive vaccine compliance.

## Data Availability

Data are available from the corresponding author on reasonable request.

## Abbreviations

ECDC: European Center for Disease Prevention and Control
LEA: Essential Care Levels of Assistance
MMR: Measles, Mumps and Rubella
PCV: Pneumococcal conjugate vaccine 13
pHCPs: Pediatric Health Care Professionals
PN: Pediatric Nurses
PNPV: National Immunization Prevention Plan
PPV: Pneumococcal polysaccharide vaccine 23
SIAIP: Italian Society of Pediatric Allergy and Immunology
WHO: World Health Organization
